# Steroids, Pregnancy and Fetal Development

**DOI:** 10.3389/fimmu.2019.03017

**Published:** 2020-01-22

**Authors:** Maria Emilia Solano, Petra Clara Arck

**Affiliations:** Department for Obstetrics and Fetal Medicine, University Medical Center Hamburg-Eppendorf, Hamburg, Germany

**Keywords:** glucocorticoids, progesterone, hormone receptors, pregnancy pathophysiology, fetal programming

## Abstract

Maternal glucocorticoids critically rise during pregnancy reaching up to a 20-fold increase of mid-pregnancy concentrations. Concurrently, another steroid hormone, progesterone, increases. Progesterone, which shows structural similarities to glucocorticoids, can bind the intracellular glucocorticoid receptor, although with lower affinity. Progesterone is essential for the establishment and continuation of pregnancy and it is generally acknowledged to promote maternal immune tolerance to fetal alloantigens through a wealth of immunomodulatory mechanisms. Despite the potent immunomodulatory capacity of glucocorticoids, little is known about their role during pregnancy. Here we aim to compare general aspects of glucocorticoids and progesterone during pregnancy, including shared common steroidogenic pathways, plasma transporters, regulatory pathways, expression of receptors, and mechanisms of action in immune cells. It was recently acknowledged that progesterone receptors are not ubiquitously expressed on immune cells and that pivotal features of progesterone induced- maternal immune adaptations to pregnancy are mediated via the glucocorticoid receptor, including e.g., T regulatory cells expansion. We hypothesize that a tight equilibrium between progesterone and glucocorticoids is critically required and recapitulate evidence supporting that their disequilibrium underlie pregnancy complications. Such a disequilibrium can occur, e.g., after maternal stress perception, which triggers the release of glucocorticoids and impair progesterone secretion, resulting in intrauterine inflammation. These endocrine misbalance might be interconnected, as increase in glucocorticoid synthesis, e.g., upon stress, may occur in detriment of progesterone steroidogenesis, by depleting the common precursor pregnenolone. Abundant literature supports that progesterone deficiency underlies pregnancy complications in which immune tolerance is challenged. In these settings, it is largely yet undefined if and how glucocorticoids are affected. However, although progesterone immunomodulation during pregnancy appear to be chiefly mediated glucocorticoid receptors, excess glucocorticoids cannot compensate by progesterone deficiency, indicating that additional und still undercover mechanisms are at play.

## Introduction

In order to support mammalian pregnancies, a myriad of adjustments in maternal physiology takes place. For example, maternal immune responses are tightly regulated to prevent inflammatory responses and rejection of alloantigens expressed on fetal tissues (1, 2). The maternal immune adaptations to pregnancy are pivotally modulated by endocrine signals. These signals include the pronounced rise of sex hormones such as progesterone and estradiol. Progesterone is essential for the establishment and continuation of pregnancy (3). Progesterone not only plays multiple immunomodulatory functions (4), but also it supports uterine receptivity and quiescence (3, 5). Additionally to sex steroids, maternal glucocorticoids dramatically increase over the course of pregnancy in order to meet the increasing energy demands (6). Glucocorticoids are potent activators of GR, and this activation has pleiotropic effects on immune cells (7, 8). However, the molecular mechanisms underlying how glucocorticoids contribute to the maternal immune adaptation to pregnancy and the interplay between glucocorticoids and sex hormones such as progesterone remain largely unclear.

Intriguingly, although progesterone is generally acknowledged to promote maternal immune tolerance to alloantigens derived from the conceptus, progesterone receptors are not ubiquitously expressed on immune cells (9). Light was shed into this enigma only very recently, when it was identified that pivotal features of progesterone induced- maternal immune adaptations to pregnancy are mediated via the glucocorticoid receptor (9, 10). Hence, in the present review manuscript, we aim to revisit the current evidence about the synthesis and interplay between glucocorticoids and progesterone during pregnancy, their impact on the immune system and consequences for pregnancy maintenance and fetal development.

## Progesterone and Glucocorticoid Synthesis, Regulation and Receptors During Pregnancy

### Progesterone and Glucocorticoid Receptors in Immune Cells

Both, progesterone and glucocorticoids, are significantly involved in the regulation of immune responses (4, 7, 11). The structural similarities between glucocorticoids and progesterone raise the intriguing concept of mutual, interrelated as well as individual pathways elicited by these hormones. This concept gains relevance in the context of pregnancy, where disequilibrium between these steroids is related to altered maternal immune responses and pathological pregnancy outcomes (2, 7).

The genomic effects of progesterone and glucocorticoids are mediated by the intracellular progesterone and glucocorticoid receptors (PR and GR), which belong to a subfamily of the nuclear receptor superfamily (4, 7, 12). Upon binding to ligands, PR and GR translocate to the cell nuclei, where they interact with specific regions of the DNA to act as transcription factors that modulate gene expression (7, 11, 12). Despite the high amino-acid identity between PR and GR (12), their steroid binding affinities, expression patterns, and target genes differ remarkably, as summarized in Table 1.

**Table 1:** Comparison between general features of the progesterone and glucocorticoid receptors.

	**Progesterone receptor**	**Glucocorticoid receptor**	**Membrane progestin receptors (mPR)**	**Progesterone receptor membrane components (PGRMC)**
Genes	NR3C3	NR3C1	PAQR 5-9 (progestin and adipoQ receptor)	PGRMC1 and PGRMC2
Isoforms/subtypes	PRA and PRB isoforms	Multiple isoforms, including variants of GRα, GRβ, GRγ, GRA, GRB and GRP (13)	mPRα (PAQR7), mPRβ (PAQR8), mPRγ (PAQR5), mPRδ (PAQR6) and mPRε (PAQR9)	PGRMC1 and PGRMC2
Relative binding affinity^*^	Progesterone: 100% (14–16) other progestogens: 1–46% (16)	Progesterone and other progestogens: 1–6% (14) or 40% (15)	progesterone: 100% (17)	progesterone: 100%
	Corticosterone: 2.6% (16) Dexamethasone: 0.2% (15)	Corticosterone: 85% (16) Dexamethasone: 100% (16)	glucocorticoids: 0–26% (17, 18)	glucocorticoids: low affinity (19)
Expression in immune cells	Limited to specific cell lineages (9, 20, 21)	+++ (9, 20)	++ (20, 22) or undetermined	++ (22) or undetermined
Uterus	+++ (23)	++ (23, 24)	++ (22, 25)	+++ (26)
Genomic pathways	Dimers act as transcriptions factors by binding progesterone response elements	Gene transactivation or transrepression through DNA and/or transcription factor binding (27)	–	–
Non-genomic pathways	Monomers activate MAPK pathways through Src-kinase (28)	Binding to membrane receptors (27) and signaling through cytoplasmic ligand-bound GR and chaperone proteins (8)	Still controversial. Pathways may involve G-proteins and modulation of adenylyl cyclase activity (4, 18, 29)	Multiple intracellular signaling pathways, e.g., interacts with EGFR, ERK1, casein kinase 2, and PDK (30)

* *Compared to the respective ligand with higher affinity*.

The Nr3c1 gene encoding for GR is expressed in most tissues of the organism, and virtually in all cells of the immune system (31, 32). Glucocorticoids can bind the GR with high affinity to elicit genomic but also non-genomic pathways in immune cells (7, 33). Importantly, promiscuous binding of progesterone to GR has also been observed in a number of settings, particularly in *in vitro* models (9, 14). Due to alternative splicing and alternative translation initiation sites, many isoforms of the GR have been described (7, 13). These isoforms are also present in immune cells and associated with diverse translational activities or binding to glucocorticoids (7, 34). However, it remains unknown whether GR isoforms are affected during pregnancy or if they have differential affinity for progesterone. Indeed, as detailed in Table 1 most progestogens have only very limited affinity to glucocorticoid receptor compared to glucocorticoids (14–16, 34).

The Nr3c3 gene encodes for two PR isoforms, PRA and PRB (35). Both PR isoforms have differential transcriptional activity and are predominantly found in mammary gland and in the female reproductive tissues, such as the ovary and uterus (23, 35). Overall, the presence of PR in immune cells is a matter of controversy. Although a direct effect of progesterone on e.g., T cells during pregnancy has long been proposed (36–39), recent findings based on RT-qPCR approaches aiming to detect PR on distinct immune cell subsets failed to confirm the expression of PR in e.g., T and NK cells (9, 20, 40, 41). Promiscuous binding of PR by glucocorticoids has been reported, although there is no consensus on the reported relative binding affinities compared to progesterone (14, 15).

Besides the PR, progesterone can elicit non-genomic actions by binding to G-protein coupled membrane progestin receptors (membrane progesterone receptors: mPR) and the so-called progesterone receptor membrane components (PGRMC) [reviewed in (4)]. Among them, mPRalpha/PAQR7 and mPRbeta/PAQR8 as well as PGRCM1 and 2 are present in T cells (20, 29) and mPRalpha is expressed in particular fractions of circulating Tregs (42). Hence, these pathways may explain some of the effects of progesterone on immune cells. Of note, information on glucocorticoid binding to mPRs is ambiguous [(18), Table 1), whilst glucocorticoid binding to PGRMCs has been described, albeit with low affinity (19).

Taken together the close structural similarities and the cell-restricted expression of receptors, progesterone and glucocorticoids may act on immune cells via non-genomic pathways as well as by likely binding to GR rather than to PR. Due to their high levels during pregnancy, it seems plausible that both progesterone and glucocorticoids act on GR to trigger immunoregulatory signals. This will depend on the bioavailability of the steroids, which varies across pregnancy according to their synthesis, the amount of carrier proteins limiting the free steroids reaching the tissues as well as from the metabolism or exclusion of these steroids from the target cells.

### Bioavailability of Progesterone and Glucocorticoids During Pregnancy

Steroid synthesis such as in the case of progesterone and glucocorticoids consists of the conversion of cholesterol as a substrate through a series of enzymatic reactions, to produce structurally interrelated products. This process is tightly regulated by the tissue- and cell-specific expression of steroidogenic enzymes (43).

For example after ovulation the ovarian follicular cells that support the maturation of the oocyte undergo the so-called luteinization process to form the corpus luteum. During luteinization, the expression of genes and proteins that mediate progesterone synthesis is prominently upregulated (44). In mice and other mammals, the corpus luteum largely accounts for the significant *de novo* synthesis of progesterone during the entire duration of pregnancy. Here, progesterone concentration in the blood increases until mid-late pregnancy, when it gradually starts decreasing (45). This progesterone deficiency is considered as an upstream event triggering parturition in mice (46). In humans, the placenta expresses the enzymes involved in progesterone production and commences steroidogenic synthesis at gestation weeks 7–9, following the initial ovarian progesterone synthesis (47). Progesterone levels continuously rise until reaching a plateau in the last weeks of pregnancy (48). A progesterone decline at late gestation does not occur in humans and it has been suggested that parturition results from a functional progesterone deficiency occurring at myometrial and other uterine tissues (4, 49). Here, differential expression of progesterone receptor isoforms may allow for progesterone-induced cervical relaxation during parturition (49), hereby promoting the delivery of the human fetus (50, 51).

It is well-known that glucocorticoids are largely produced in the adrenal cortex, where they exhibit circadian and ultradian rhythms (4). Maternal glucocorticoids rise dramatically during pregnancy, e.g., during late murine pregnancy, glucocorticoids reach an ~20-fold increase compared to mid-pregnancy concentrations (6). In humans, cortisol, the main glucocorticoid, also increases dramatically during pregnancy, reaching ~350 ng/ml serum on week of gestation 26 (52). Thereafter, cortisol remains relatively stable until parturition, when it is strongly upregulated (52). In women, corticotrophin releasing hormone (CRH) is produced by the placenta to further stimulate adrenal glucocorticoid production (53) pinpointing the critical relevance of glucocorticoid synthesis during pregnancy.

The actions of these high levels of progesterone and glucocorticoids are limited by their binding to plasmatic carrier proteins (54). Only the “free” fractions of progesterone and glucocorticoids are considered to be able to bind receptors to exert biological functions, e.g., after diffusing inside the target cells (54). Corticosteroid-binding globulin (CBG) transports around 75–80% of plasma glucocorticoids, thereby critically limiting the abundance of free glucocorticoids available to cells (55). Despite a pronounced increase of CBG levels and binding capacity throughout pregnancy (6), 5–6% of the total cortisol remains free (56). Hence, the absolute concentration of free glucocorticoids increases during pregnancy (56). In contrast, both the fraction of free progesterone and its total concentration increase throughout pregnancy (57). Progesterone only partially binds CBG with four times lower affinity as glucocorticoids. Instead, approximately the 80% of plasma progesterone primarily binds albumin (54).

The availability of steroid hormones can be additionally reduced by their intracellular metabolism. However, physiological expression of 11β-hydroxysteroid dehydrogenase type 2 capable of metabolizing glucocorticoids into inactive forms (6) is largely negligible in human or mouse immune cells (58) and potential modulation during pregnancy remains to date unexplored. Moreover, the progesterone-metabolizing enzyme 20α-hydroxysteroid dehydrogenase (Akr1c18) was shown to be highly expressed in thymocytes and initially considered as a marker for mature T cells (59, 60). However, data available to date seem ambiguous, as Akr1c18 is not listed when searching gene-expression database for immune cells (32). Hence, the significance or role of the expression of 20α-HSD or 11β-HSD in lymphocytes and possibly also myeloid cells is still unknown.

Moreover, Abcb1a and Abcc1 efflux transporters, members of the ATP binding cassette (ABC) transmembrane transporters family can actively exclude intracellular glucocorticoids hereby limiting their activity e.g., in mouse placenta (6). Abcb1a and Abcc1 (also known as Mdr1 and Mrp1) are differentially expressed in immune cells such as T lymphocytes (61) and Abcb1a deficiency was associated to decreased generation of Tregs *in vivo* and *in vitro* mouse models (62). Remarkably, progesterone and other progestogens are potent inhibitors of Abcb1a function (63), mechanism that may act synergistically with the high levels of glucocorticoids to further promote glucocorticoids actions during pregnancy.

Taken together existing published data on progesterone and glucocorticoids levels as well as their binding to plasma proteins during human pregnancy, it becomes evident that early pregnancy consists in a period of high progesterone and low glucocorticoid availability. In contrast, both free progesterone and glucocorticoids increase throughout pregnancy and are found at comparable concentration ranges in late pregnancy (48, 57). Hence, while a large body of evidence supports that steroid driven immune modulation relies mainly on progesterone at the beginning of pregnancy it is tempting to hypothesize that in later stages, glucocorticoids with high affinity for GR gain relevance in sustaining maternal immune tolerance. In this context, the regulation of progesterone and glucocorticoids bioavailability by expression of specific metabolizing enzymes and exclusion transporters in immune cells during pregnancy remains still unknown.

### Modulation of Steroids by External Factors

**Figure 1:**
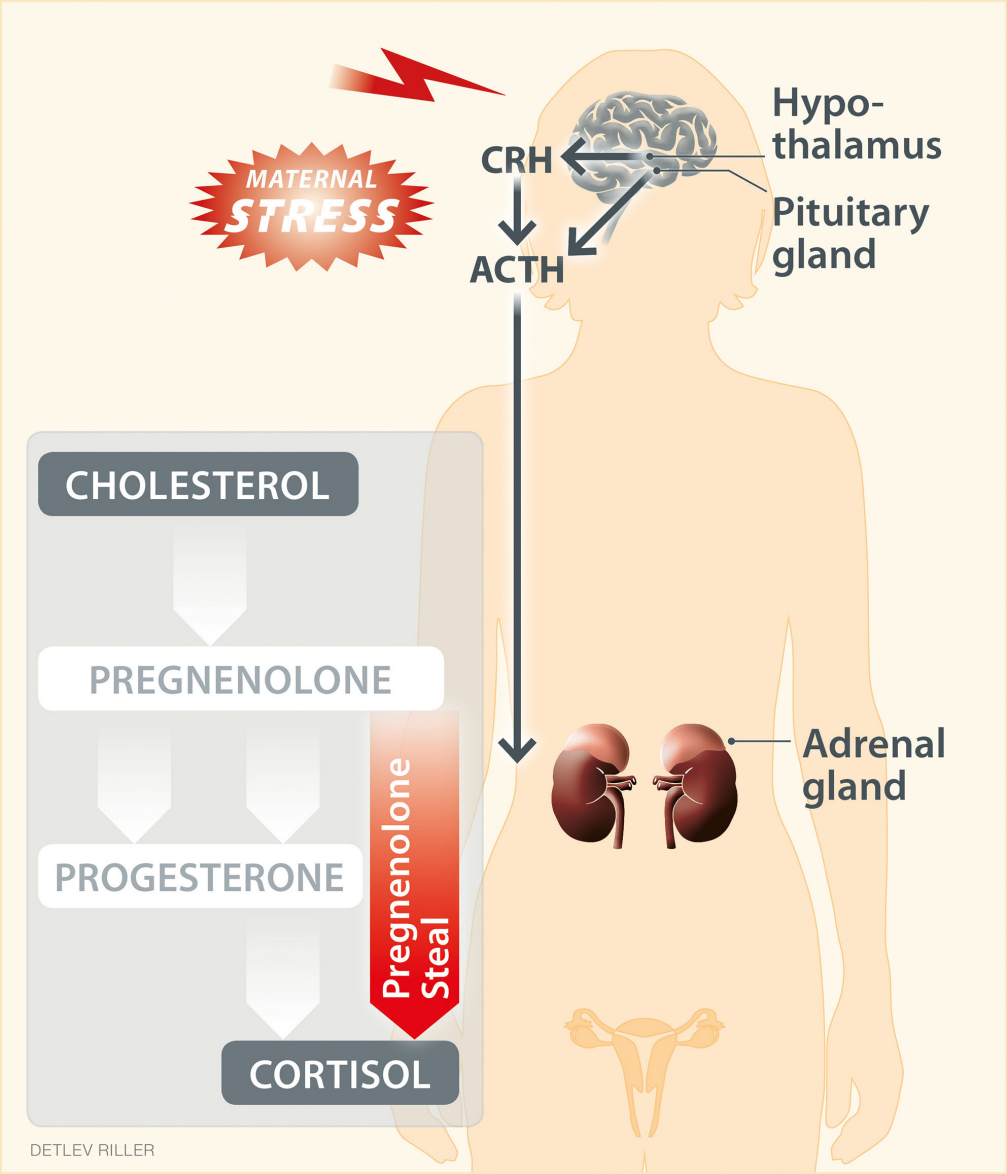
“Pregnenolone steal” or how high stress perception may drive the depletion of progesterone. High stress perception activates the hypothalamic–pituitary–adrenal axis, resulting in the respective secretion of corticotropin-releasing hormone (CRH), adrenocorticotropic hormone (ACTH) and cortisol, the main glucocorticoid in humans. Moreover, stress can affect steroidogenesis in peripheral tissues. Steroidogenesis refers to the transformation of cholesterol into steroid hormones through a serious of steps. Here, the intermediate pregnenolone is a precursor of most steroid hormones, including progesterone and cortisol. Upon stress, the elevated synthesis of cortisol may reduce (“steal”) the pregnenolone available for the synthesis of down-stream hormones other than cortisol. This hypothetical scenario provides an explanation for the impaired progesterone production in response to stress.

The availability of steroid hormones during pregnancy, but also unrelated to reproduction, can be dramatically modulated by external factors. One key example is the exposure to stress, commonly described as a high perception of stress. It is well-established that stressful stimuli trigger the activation of the hypothalamic–pituitary–adrenal (HPA) axis, which results in secretion of glucocorticoids by the adrenal glands (Figure 1). Although this neuroendocrine response is gradually attenuated across pregnancy (53), stressful stimuli can still elicit the secretion of glucocorticoids in mouse and humans (6, 64). Concomitantly, stress challenges reduce progesterone levels during pregnancy in mammals (65–68). This could result from impaired steroidogenesis in the ovary, e.g., due to poor stimulation by placental lactogens (68). Stress-induced glucocorticoids may directly influence progesterone synthesis, as GR is also expressed in the ovary, where depending on the experimental conditions they have been shown to stimulate or inhibit steroidogenesis (69, 70).

Moreover, progesterone and glucocorticoids share common steroidogenic pathways and precursors, such as *cholesterol-derived pregnenolone* (Figure 1). Hence, a hypothesis for the depletion of progesterone as a result of glucocorticoid production in response to high stress perception has been proposed (71). This hypothesis is referred to as “pregnenolone steal” (71) and supports that the elevated synthesis of cortisol caused by stress depletes (“steals”) the availability of pregnenolone for the synthesis of down-stream hormones other than cortisol, which subsequently may also impede the synthesis of progesterone. This hypothesis requires confirmation also in the context of pregnancy. The conversion of cholesterol to cortisol occurs in the mitochondria of steroidogenic tissues, best described for the adrenal cortex, but also for various other tissues, including primary lymphoid organs, intestine, skin and brain (72, 73). If cortisol synthesis could also be induced e.g., by stress in tissues such as the ovaries and placenta, it could theoretically result in a reduction of the precursors available to produce progesterone during pregnancy. Interestingly, in ovary, the main site of progesterone synthesis in early human pregnancy, the specific enzymatic machinery for glucocorticoid production has already been described (74), and it remains to be confirmed whether the pregnenolone steal may indeed impede ovarian progesterone synthesis in response to stress.

## Immune Pathways Mediated by Progesterone and Glucocorticoids

Antigenic disparity between the mother and the fetus is not only tolerated by the maternal immune system, but also promotes placental and fetal growth in mice (75). Understanding the mechanisms through which maternal immune tolerance toward fetal antigens is maintained is not only critical to decipher how survival of species is ensured. Such insights also allow shedding light on the pathogenesis of pregnancy complications. The collapse of maternal immune tolerance can become evident as cytotoxic responses at the feto-maternal interface and subsequent fetal loss (21, 76, 77) or impaired placental and fetal development (68, 78).

To date, a wealth of data highlights that high levels of progesterone are critically required to switch the maternal immune responses toward tolerance [e.g., discussed at length in (4)]. Progesterone promotes a tolerogenic profile on innate immune cell subsets, such as macrophages and dendritic cells, which is essential for successful uterine tissue remodeling and pregnancy maintenance (1–3). For example, *in vitro* stimulation with progestogens induces maturation of macrophages with M2 profile (79), and prevents the differentiation of dendritic cells toward a mature phenotype (80). A progesterone-mediated modulation of the adaptive immune responses has also been investigated in *in vivo* and *in vitro* models. Here, progesterone supports the expansion and suppressive function of Tregs during pregnancy, the skew toward an anti-inflammatory cytokine profile and suppression of CD8^+^ T cell cytotoxicity (20, 68, 81–83).

Despite the availability of PR and GR specific pharmacological agonists and antagonists (Table 1), experimental interventions during pregnancy employed most often progesterone as agonist or the antagonist RU486, both of which can bind PR and GR. Hence, these approaches do not allow differentiation between the individual effects of progesterone or glucocorticoids on distinct immune cell subsets, which greatly limits to understand the individual role of hormones or cell subsets in maintaining pregnancy. Such limitation can now be easily overcome by the use of mice with targeted deletion of certain hormone receptors on distinct immune cell subsets. In fact, recent evidence revealed that the targeted deletion of PR on dendritic cells in mice promotes a non-tolerogenic, mature phenotype of dendritic cells, along with the failure to generate CD4^+^ Treg and CD8^+^CD122^+^ Treg cells and impaired placental and fetal development (78). Also targeted gene deletion of the GR on T cells in mice pinpoints that GR and not PR is an upstream promotor of Treg expansion during pregnancy. *In vitro* approaches further support that GR mediates the expansion of T regulatory cells by selective induction of apoptosis in conventional T cells (9, 10). These mechanisms are at play during pregnancy, as in a mouse model of experimental autoimmune encephalomyelitis, GR deletion in T cells prevented pregnancy-induced expansion of T regulatory cells, as well the corresponding mitigation of autoimmunity (9).

In this context, functional analyses of the contribution of progesterone signaling through mPRs and PGRMC to immune regulation during pregnancy remain still largely elusive. To date, accumulating *in vitro* evidence highlights the importance of these non-genomic pathways e.g., on T cell responses (20, 29, 84).

Besides the direct hormone-steroid receptor interaction, progesterone can indirectly affect immune responses. Uterine and placental expression of the PR promotes the local expression of immunomodulatory molecules, such as progesterone-induced blocking factor (PIBF), galectin-1 (Gal-1) (41, 83), and heme oxygenase 1 (Hmox1) (68). These potent immunomodulators are critical for the establishment and continuation of pregnancy, as shown in mouse models and human pregnancies (41, 68, 83, 85, 86). For example, PIBF can enhance the synthesis of Th2 cytokines and dampens NK cell cytotoxicity (41) whereas Gal-1 induces a tolerogenic phenotype in dendritic cells, which results in Treg expansion (81). In turn, the enzyme Hmox1 supports the generation of CD8^+^CD122^+^ regulatory T cells that during pregnancy promote placental vascularization and fetal growth (68). Pathways involved in progesterone-mediated promotion of pregnancy maintenance may also include the epigenetic silencing of key T cell-attracting inflammatory chemokine genes in decidual stromal cells, as observed in mice upon progesterone stimulation (87). This epigenetic silencing of chemokine genes can subsequently suppress the accumulation of anti-fetal effector T cells in the decidua, hereby reducing the risk for fetal loss.

Some of progesterone-induced pathways in the uterus could also be mediated by GR. In fact, although glucocorticoids seem to be dispensable during early pregnancy (88) uterine GR expression is critical to ensure successful pregnancy. Evidence arising from transgenic mice shows that a targeted deletion of GR in the uterus results in subfertility, excessive inflammation and altered immune cell recruitment during decidualization (23).

In the light of these recent observations, an upstream role of GR in pregnancy induced immune tolerance is underscored, while new questions on the roles of progesterone and glucocorticoid non-genomic pathways appear. These concepts challenge previous notions on processes taking place during pregnancy and invite not only to revisit former data but also to advance in the research of these endocrine-immune mechanisms from this novel perspective. Of note, a number of technical tools to discriminate the receptor-specific pathways are to date available (Table 2) and promise exciting progress in the research in the field.

**Table 2:** Salient technical tools available to discriminate steroid receptor-specific pathways.

	**Progesterone receptor**	**Glucocorticoid receptor**	**Membrane progestin receptors (mPR)**	**Progesterone receptor membrane components (PGRMC)**
Selective agonists	20α-dihydrodydrogesterone (DHD) (89)	Dexamethasone, betamethasone (15), ZK209614 (90)	progesterone conjugated to BSA (50)	
Antagonist	non-selective: RU-486		–	–
	selective: ZK98299 (91), Ulipristal acetate (92), Org31710 (93)	selective: RU-43044 (94)		
Mouse models for cell specific depletion	Pr^fl/fl^ (21)	Gr^fl/fl^ (9, 10)	–	Pgrmc1^fl/fl^ and Pgrmc2^fl/fl^ (30)

## Impact of Progesterone and Glucocorticoids on Pregnancy Outcome and Maternal Immune Response

**Figure 2:**
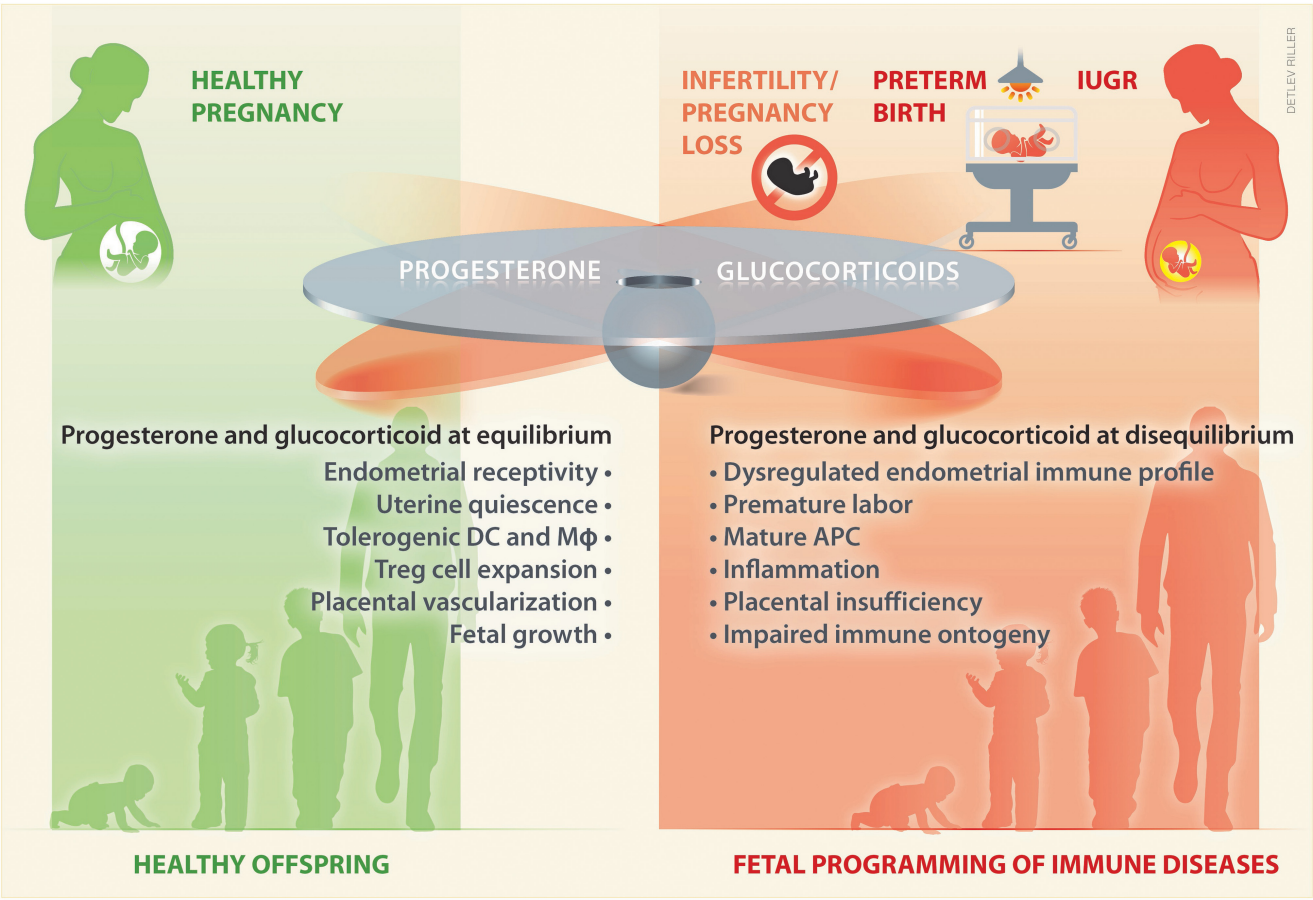
During pregnancy a tight balance between glucocorticoids and progesterone may take place. An equilibrium between these hormones ensures adequate levels to sustain uterine receptivity and quiescence, as well as a tolerogenic immune profile, which pivotally promotes placental vascularization and a healthy fetal growth. In contrast, a disequilibrium in progesterone and/or glucocorticoids may fail to sustain pregnancy, and underlie an altered intrauterine immune profile, prone to inflammation, which leads to placental insufficiency and poor fetal growth. Such a disequilibrium may play an upstream role in women suffering from infertility or from pregnancy complications, such as early pregnancy loss, preterm birth, and IUGR. Impaired fetal growth and altered prenatal exposure to glucocorticoids influences the fetal immune ontogeny, which may result on fetal programming of immune disease in the offspring. DC, dendritic cells; M*ϕ*, macrophages; APC, antigen presenting cells; IUGR, intrauterine growth restriction.

Given the shared steroidogenic pathways and transport of progesterone and glucocorticoids as well as their widespread crosstalk in immune cells and reproductive tissues, it is tempting to speculate that a tight equilibrium between these steroids underlies healthy pregnancy and fetal development (Figure 2). As discussed below, this equilibrium can be disrupted with consequences for the establishment or continuation of pregnancy or affecting the developing offspring (Figure 2). Hence, progesterone and glucocorticoids appear as attractive pharmacological treatments, e.g., that could restore maternal immunotolerance, and they are often supplemented to women at risk for pregnancy complications.

### Progesterone, Infertility, and Early Pregnancy Loss

Worldwide, around 10% of couples experience fertility problems, whereby male and female factors almost equally account for these incidences. Interestingly, the overall burden of female infertility has remained similar over the last 2 decades, despite the progress in assisted reproductive techniques (95). Besides infertility, early pregnancy loss clinically defined as spontaneous miscarriage before the week 20 of gestation occurs in 10–15% of healthy women (96). A large fraction of spontaneous miscarriages is due to unknown etiologies, in which immune maladaptations, e.g., in response to environmental factors (97), are suspected to play a critical role.

Progesterone insufficiencies and related inability to mount an appropriate immune response favoring embryo implantation has been frequently put forward to explain these incidences. However, to date, the high variability in progesterone secretion and the limitation to measure glucocorticoids in clinical routine hinder the diagnosis of progesterone deficiency or glucocorticoid imbalances during normally progressing pregnancies as well as pathologies such as infertility and spontaneous miscarriage (98, 99). Given the soaring levels of steroid hormones occurring during pregnancy, endocrine interventions have been frequently used in couples suffering from infertility or pregnancy losses. Infertile women orally treated with the progestogen Dydrogesterone, which shows a high affinity for the PR, had higher birth rates compared to treatment with vaginal micronized progesterone (100). However, the potential modulation of the maternal immune response by these treatments has not been tested.

Similar to the infertility trial described above, treatment with oral Dydrogesterone also reduced the risk in women with a history of recurrent pregnancy loss, whereas treatment with vaginal micronized progesterone failed to reduce the abortion risk (101). In this study, cytokine levels were tested and significantly differed between women with recurrent pregnancy loss who were assigned to the different treatment arms, which limits the analyses of treatment effects on immune responses. Comparably, progesterone withdrawal or blockage results in fetal loss in mammals (83, 102, 103) and the PR and GR antagonist RU486 is effectively employed to terminate human pregnancies (104, 105).

Insights into the mechanisms underlying the pregnancy protective effects induced by oral progestogens are highly desirable. Considering that vaginal administration of micronized progesterone did not improve implantation success in infertile patients and failed to reduce the abortion rate, it can be speculated that the oral route of application increase systemic progestogen levels to the degree required in order to initiate the pregnancy-protective effects on the maternal immune system.

Additional evidence for an upstream role of progesterone in ameliorating the risk for pregnancy pathologies arise from more recent studies on progestogens supplementation during early pregnancy (3, 106, 107). Reduced progesterone, e.g., due to luteal insufficiency or stress may influence maternal tolerance toward fetal antigens and result in fetal loss (108, 109). Despite the wealth of information on the interaction between progesterone and the immune response, very little insights into the causal relationship between altered hormones levels, collapse of the maternal immune tolerance and subsequent pregnancy loss are available to date, which should be addressed in future trials.

Due to their potent immune regulatory capacity, glucocorticoids appear as a potential therapeutic option in women suffering from with repeated idiopathic embryo implantation failure. Corticoid therapy is becoming an important medication for patients with history of repeated implantation failures (RIF) after IVF/ICSI and at least a proportion of the patients respond to such intervention (110). Indeed, emerging data accumulated in small group of patients with increased numbers of NK cells in the endometrium suggests potential beneficial effects of corticosteroid therapy (111) as intrauterine perfusion of dexamethasone reduced NK cell frequencies and resulted in successful pregnancy (112). Of note, the safety of glucocorticoid administration during pregnancy has not yet been completely clarified (111, 113) and concerted efforts need to be devoted to identifying patients that can specifically benefit from corticosteroid therapies (114).

### Preterm Labor

Rates of prematurity are currently on the rise, not only in developing countries or countries in transition to development, but also globally (115). Consecutively, preterm birth is the main reason for newborn death worldwide and a major contributing factor to poor offspring's health. Progress has been made to predict the risk for preterm birth, but its etiology is still enigmatic. In the context of preterm birth, the importance of the maternal immune system is increasingly recognized. Term labor is initiated by complex pathways, which include the up-regulation of inflammatory signals (116). Pilot data suggest that the collapse of maternal immune adaption and a premature activation of inflammatory pathways trigger labor prematurely (117). Here, it remains to be demonstrated whether the up-regulation of inflammatory signals follows a functional progesterone withdrawal. In fact, vaginal progesterone application has been demonstrated to decrease the risk of preterm birth and to improve perinatal outcomes in singleton gestations with a short cervix in humans, suggesting that progesterone ensures uterine quiescence in cervical tissue (115). Very recently, it has been demonstrated that treatment with progesterone may be a strategy to prevent preterm labor/birth and adverse neonatal outcomes by attenuating the proinflammatory responses at the maternal-fetal interface and cervix induced by T cell activation (24).

Similar to PR, the myometrium expresses GR, although at lower levels (118), and some of anti-inflammatory progesterone actions in this tissue, e.g., COX-2 or IL-1β repression may be also mediated by GR (23, 93). At term labor glucocorticoids are potently triggered (52). However, there are no reports on beneficial effects of glucocorticoids on the maternal outcomes, e.g., on women that received antenatal steroid therapy for fetal lung maturation. Altogether, potential implications of maternal glucocorticoids on the modulation in preterm labor are not yet clearly established.

### Intrauterine Growth Restriction

Intrauterine growth restriction (IUGR) refers to suboptimal fetal growth, a condition that affects 3–10% of pregnancies (119). IUGR may result from placental insufficiency, e.g., due to impaired uterine or placental vascularization. Progesterone can promote uterine and placental vascularization by diverse pathways. For example, progesterone upregulates the VEGF homolog placental growth factor (PlGF) (120), which is expressed by trophoblast and uterine NK cells (121, 122). PlGF promotes NK cytokinesis and consequently decidual spiral arteries remodeling during early pregnancy and labyrinth vascular branching in mid to late murine pregnancy (122). Indeed, it is well-accepted that uterine NK cells (122) promote pregnancy related uterine vascular changes through pathways including the secretion of cytokines such as IFN-γ and IL-17. IFN-γ affects uterine vasculature and stromal gene expression, which leads to vessel instability and facilitates remodeling of decidual arteries (123). Recently, it was also proposed that progesterone and estradiol trigger apoptosis in neutrophils, which transfer proteins to T cells. These “neutrophil-induced T” (niT) cells upregulate regulatory markers and promote vessel growth *in vitro* through IL-17 and VEGF expression (124).

Moreover, in a mouse model of mid-gestational stress we observed that reduced progesterone was associated to epigenetic changes in the placenta that resulted in decreased heme oxygenase-1 (Hmox-1) expression and IUGR. These changes were caused by an increase of cytotoxic CD8^+^ T cells producing inflammatory cytokines. This inflammatory surge was unopposed by CD8^+^CD122^+^ T regulatory cells. Notably, supplementation of progestogens mitigated the IUGR by restoring Hmox-1 expression as well as suppressing inflammation (68).

Intriguingly, stress-induced intrauterine inflammation takes place in an environment rich in glucocorticoids (6, 68). Glucocorticoids can affect placental gene expression and growth (6, 125), with consequences in the nutrition and gas exchange with the fetus. These effects together with potential fetal excessive glucocorticoid exposure are hypothesized to underlie intrauterine growth restriction i.e., in the case of maternal dietary protein restriction, or stress [reviewed in (8)].

Together these observations provide evidence that the functions of progesterone and glucocorticoids are not exchangeable and that a regulated balance is required in the uterus to promote fetal growth.

### Prenatal Exposure to Excess Glucocorticoids: Fetal Programming of Postnatal Immunity

During late gestation, glucocorticoids are required to ensure structural and functional organ maturation in the fetus (126, 127). However, prenatal exposure to glucocorticoid surges is detrimental for fetal growth and may hold significant consequences for postnatal physiology (8). Fetal glucocorticoid excess can be induced e.g., by antenatal steroid treatments in the case of risk for preterm birth (128). Additionally, antenatal glucocorticoid exposure is proposed to underlie a number of conditions, such as maternal malnutrition (129), stress (6), and infection (130). In mice, prenatal stress and the consequent fetal glucocorticoid excess resulted in intrauterine growth restriction (IUGR) particularly in female offspring (6). These observations could be explained by sex specific stress responses at the placenta, which limits the transfer of maternal glucocorticoids to the fetus. Indeed, placentas from female offspring failed to upregulate placental protective mechanisms, such as 11β-HSD2 and ABC transporters in response to antenatal stress, whereas these protective mechanisms prevented glucocorticoid excess in male fetuses (6).

Growing evidence underscores a role of prenatal glucocorticoid exposure in offspring's immune ontogeny and impaired postnatal immunity (131, 132). These effects could be multifactorial, including indirect and direct effects in the immune system (8). For instance, prenatal stress or glucocorticoid excess can result in disarrangements in the HPA [reviewed e.g., in (133)]. Generally, it is widely accepted that postnatal HPA hypoactivity follows prenatal stress exposure (134). Metabolic disarranges in offspring exposed to prenatal stress or glucocorticoids have also been observed and include the programming of a thrifty metabolic phenotype (135). Both postnatal HPA and metabolism may affect postnatal immune responses. Remarkably, premature exposure to glucocorticoids may also affect the developing fetal immune system [reviewed in (8)]. For example, antenatal steroid treatment resulted in newborns with impaired immunity (136) e.g., due to poor neutrophil (137) and T cell (138) responses.

## Final Remarks

Recent data emerging from mice carrying cell specific gene deletions underscore that pathways downstream the GR in immune cells are critically involved in promoting immune tolerance during pregnancy (9, 10). As until recently this tolerance was considered to be primary modulated by signaling through the intracellular PR, these novel observations invite to reexamine aspects of endocrine immune regulation during pregnancy. In early pregnancy such GR-mediated pathways are likely elicited by high levels of progesterone. However, glucocorticoids with high affinity for GR outpace progesterone levels in mid-late stages of gestation. Simultaneously the maternal inflammatory load intensifies due to the cumulative exposure to antigens derived from the conceptus (139). Whether this glucocorticoid predominance translates into a chief immunomodulatory role remains unknown and requires empirical validation. Taken together the here summarized data, it is tempting to anticipate the proximity of a paradigm shift with regards to immune-endocrine responses during pregnancy e.g., related to signaling pathways or potential therapies to promote immune tolerance during pregnancy.

Of note, glucocorticoids and progesterone appear to be present in a tight equilibrium during pregnancy. Even subtle disruptions of this equilibrium may have significant consequences for pregnancy progression and fetal development (8, 68) (Figure 2). However, detailed information on their modulation and potential associations to inflammatory mechanisms taking place in the context of pathological pregnancies remain largely elusive. This is at least partly due to the fact that progesterone and glucocorticoids are not routinely assessed during pregnancy. Such assessments could refine the identification of women that can benefit from endocrine therapies to achieve or support pregnancy and fetal growth.

Finally, the tight crosstalk between pathways downstream progesterone and glucocorticoids could have therapeutic implications. In clinical praxis, glucocorticoids are broadly employed to reduce inflammation in pathological settings. Still, due to the side effects of their long-term use, a great body of research has attempted to find active compounds that could replace corticosteroids particularly as a chronic therapy. It could be hypothesized that progesterone could be such an alternative. For example, the mitigation of the course of multiple sclerosis in pregnant women, with an intensification of the disease activity in the postpartum period (140), suggests an upstream immunomodulatory role of pregnancy-induced hormones (9, 141). However, a recent clinical trial failed to demonstrate an effect of progestogens in preventing post-partum relapses in women suffering from multiple sclerosis (141) implying a limited efficacy of the treatment applied in this trial. Hence, despite its high clinical relevance, the empirical evidence to support the use of progestogens as a replacement for glucocorticoids remains to date sparse and requires still thorough investigation.

## Author Contributions

Both authors have made a substantial intellectual contribution to the work, and approved it for publication.

### Conflict of Interest

The authors declare that the research was conducted in the absence of any commercial or financial relationships that could be construed as a potential conflict of interest.
